# Dr. Mayfield’s Article

**Published:** 1888-08

**Authors:** 


F. E. DANIEL, M. D., EDITOR AND PUBLISHER.

THIS JOURNAL, by unanimous vote of the members, was made the Official Organ of the Austin District Medical Society.

COLLABORATORS:

*Dr. R. M. Swearingen, Austin.*
*Prof. B. E. Hadra, M. D., Galveston.*
*Prof. Geo. Cupples, M. D., San Antonio.*
*Dr. T. C. Osborn, Cleburne, Texas.*
*Dr E. J. Doering, Chicago.*
*Dr. E. J. Beall, Fort Worth.*
*Dr Odo Betz, Germany.*
*Dr. J. W. McLaughlin, Austin.*
*Dr. T. J. Tyner, Austin.*
*Prof. J. F. Y. Paine, M. D., Galveston.*
*Dr. R. H. L. Bibb, Mexico.*
*Dr. T. J. Bennett, Austin.*
*Dr. Bat Smith, Wharton.*
*Dr. E. Meierhof, New York.*
*Prof. W. B. Rogers, M. D., Memphis, Tenn.*

### DR. MAYFIELD'S ARTICLE.—THE PROFESSION AND THE PUBLIC.

Dr. Mayfield's paper in this issue of the JOURNAL, is commended to our readers, and especially to our legislators, and others in authority, as being a sound and sensible exposition of the "situation,"—of the *reasons why* the medical profession has so little influence collectively. The JOURNAL has, more than once, pointed out, that until thoroughly organized, the medical profession can never obtain recognition as an entity, and can have no influence in public matters. As the Galveston News says: "it has no official status." Until it has "official status," and is recognized as an element in politics, we can never hope to impress the Legislature with the facts so ably presented by Dr. Mayfield, nor convince them of the sincerity and purity of motive in asking for legislation to regulate the practice. The JOURNAL has discussed the subject until it is well-nigh threadbare; but it is only by persistence that we hope finally to win.

When the profession shall become completely organized,—when every respectable physician, within the broad limits of the State, shall become enlisted in the cause, and takes up arms in the war on quacks—and a petition to the Legislature voices the sentiment of a united profession—numbering perhaps three thousand of the best educated and enlightened citizens, then, and not until then, can we expect the "Bells of Cooke" to pay respectful attention to any request emanating from "the Doctors." Why—even in the little matter of asking the Legislature to set aside one room in the capitol (when fifty are vacant), for the use of the State Medical Association and local societies, and for the preservation of valuable archives, and the founding of a medical library, the petition was ridiculed, sneered at, and "Bell of Cooke" killed it, by proposing to set aside a room also for the "United Order of Bootblacks," and for the "Benevolent Association of bald-headed Barbers," (or words to that effect!)

A knowledge of these things; of the indignity offered the profession; the ignominious treatment accorded by the Nineteenth Legislature to the petitions of the State Medical Association, should arouse the pride, and awaken the indignation and resentment of every true physician in Texas. It should induce them at once to join the ranks and take up arms; to rally to the standard raised some twenty years ago by a band of devoted physicians impressed with the importance of organized efforts—a nucleus which exists to-day, but nominally only, representing the profession of the State. The Texas State Medical Association numbers 417; about ten per cent. of the physicians of the State.

But, in order to the accomplishment of the purpose, to obtain recognition and influence, to become an element in the politics of the State, to procure legislation in the interest of public health, it is not absolutely necessary that all should join the State Association. By the organization of local societies, physicians remote from the places of meeting of the T. S. M. A., can affiliate with, and be represented in the State Association. If every county were organized in this way, the question of medical legislation could and should be made an issue in local and general elections. Every member could and should exert influence with his *clientelle*, and thus the election of the "Bells of Cooke" to the Legislature could be prevented; the members of the local societies and their friends agreeing to support no man for the Legislature who would not promise to support a measure looking to the elevation and purification of the profession of medicine, and the protection of the public from the danger of quacks and impostors; they should go farther, and withhold their support from any newspaper which is so purblind or prejudiced as to oppose such measure. There are some papers which cannot, or will not, see the importance to the State, and to the future, of a preservation of medical archives, and hence *they* ridicule the petition for a room in the capitol. This is downright stupidity, or worse,—prejudice. Every paper, every scrap of information relative to epidemic or epizootic diseases, which have prevailed in Texas, historical data of their outbreak and spread, as well as of remedies employed, and measures of suppression, should be preserved for the use of the future historian and statistician ; for,—the day will come when the Legislature will see the necessity and importance of the collection and preservation of vital statistics, (and by the by, this is another subject on which legislation is needed ; and who more competent to dictate it than the medical profession?)

We repeat, nothing can be expected from the State towards the medical profession, until it is organized, and makes its influence felt in *elections*. Dr. Mayfield's paper is the slogan note of the fall campaign, and we hope to see it taken up by the profession everywhere and agitated. The people and the secular press must be undeceived as to the motives of the physicians in desiring legislation, as well as enlightened on the necessity of preventive medicine,—and shown the danger to health and happiness ("purse" and all), of a promiscous practice of medicine. The danger of epidemics are trivial, as compared to those ot an unscrupulous and ignorant pretender in medicine. The qnack must go.

